# Brain Stimulation and Group Therapy to Improve Gesture and Social Skills in Schizophrenia—The Study Protocol of a Randomized, Sham-Controlled, Three-Arm, Double-Blind Trial

**DOI:** 10.3389/fpsyt.2022.909703

**Published:** 2022-07-07

**Authors:** Victoria Chapellier, Anastasia Pavlidou, Daniel R. Mueller, Sebastian Walther

**Affiliations:** Translational Research Center, University Hospital of Psychiatry and Psychotherapy, University of Bern, Bern, Switzerland

**Keywords:** communication, social cognition, psychosis, theta burst stimulation, transcranial magnetic stimulation, intervention, cognitive remediation, schizophrenia

## Abstract

An important component of nonverbal communication is gesture performance, which is strongly impaired in 2/3 of patients with schizophrenia. Gesture deficits in schizophrenia are linked to poor social functioning and reduced quality of life. Therefore, interventions that can help alleviate these deficits in schizophrenia are crucial. Here, we describe an ongoing randomized, double-blind 3-arm, sham-controlled trial that combines two interventions to reduce gesture deficits in schizophrenia patients. The combined interventions are continuous theta burst stimulation (cTBS) and social cognitive remediation therapy (SCRT). We will randomize 72 patients with schizophrenia spectrum disorders in three different groups of 24 patients. The first group will receive real cTBS and real SCRT, the second group will receive sham cTBS and real SCRT, and finally the third group will receive sham SCRT. Here, the sham treatments are, as per definition, inactive interventions that mimic as closely as possible the real treatments (similar to placebo). In addition, 24 age- and gender-matched controls with no interventions will be added for comparison. Measures of nonverbal communication, social cognition, and multimodal brain imaging will be applied at baseline and after intervention. The main research aim of this project will be to test whether the combination of cTBS and SCRT improves gesture performance and social functioning in schizophrenia patients more than standalone cTBS, SCRT or sham psychotherapy. We hypothesize that the patient group receiving the combined interventions will be superior in improving gesture performance.

**Clinical Trial Registration:**

[www.ClinicalTrials.gov], identifier [NCT04106427].

## Introduction

Schizophrenia is characterized by negative (e.g., affective flattening, avolition, low social drive) and positive symptoms (e.g., hallucinations, agitation, delusions) (1–3), as well as, disorganized thinking (4), motor abnormalities (5–7), and impaired cognitive function (8, 9). Social cognition, which refers to psychological processes that allow for the decoding of the behaviors and intentions of others (10–12), is also impaired in schizophrenia (13). In fact, impaired nonverbal communication is a core characteristic of social cognition deficits in patients with schizophrenia. Impaired nonverbal communication in schizophrenia includes limited nonverbal social perception (14–16) and poor gesture performance (17–19). Most importantly, gesture deficits are directly associated with patients’ poor functional outcome (20). This association between gesturing and functioning is the main reason why we believe that establishing treatments, which alleviate gesture deficits are essential to improve patients’ overall functioning and quality of life (21). Such treatments can potentially facilitate societal participation. With the current trial, we want to determine whether combined brain stimulation and group therapy improve schizophrenia patients’ gesture skills and social functioning.

### Gesture Deficits in Schizophrenia

Gestures are an integral feature of communication: they support language production and transmit information on their own (22). Gesture deficits are prevalent in approximately two-thirds of patients with schizophrenia (17, 18, 23). Accumulating evidence suggests that various domains of gesturing are impaired in schizophrenia, including use of gestures (17, 18, 24, 25) and gesture perception (26). In fact, individuals with schizophrenia often use incoherent and fewer gestures (17, 27, 28) and tend to interpret gestures negatively (29, 30).

Gesture deficits in patients are associated with core schizophrenia symptoms, e.g., negative symptoms (24, 27), frontal lobe dysfunction (17, 18), formal thought disorder (23, 31) and working memory impairments (15, 24). At a neural level, gesture processing involves the praxis network, which combines motor- and speech-related brain areas (32, 33). Neuroimaging demonstrated structural and functional alterations in the praxis network in schizophrenia patients with gesture deficits (34–38). Specifically, two areas of the praxis network are linked to gesture deficits in schizophrenia: the inferior parietal lobule (IPL) and the inferior frontal gyrus (IFG) (39, 40). The IPL has been related to motor functioning, as well as cognitive and visuospatial processing (41–43). The IFG has been associated with motor functioning and language production (44).

### Interventions Improving Gesture and Social Skills

#### Brain Stimulation

Repetitive transcranial magnetic stimulation (rTMS) has the potential to (re-)train distinct neural pathways and therefore rehabilitate specific functions in multiple neuropsychiatric disorders (45). Recent studies demonstrated rTMS efficacy across different domains in patients with schizophrenia. For example, rTMS over the supplementary motor area (SMA) ameliorated motor abnormalities (46, 47) and rTMS over the inferior parietal lobe (IPL) improved gesture performance (48). Different rTMS protocols can have different effects on brain function, depending on their frequency and type of stimulation (49).

Theta burst stimulation (TBS) is an innovative rTMS protocol, which requires less time than standard rTMS. There are two types of TBS: intermittent theta burst stimulation (iTBS), which mainly has facilitatory effects and continuous theta burst stimulation (cTBS), which has inhibitory effects. A pilot study demonstrated that inhibitory cTBS over the right inferior parietal lobe (rIPL) improved gesture performance by comparing 3 rTMS protocols (including sham stimulation) in 20 patients (48). Due to interhemispheric rivalry, cTBS on the right IPL may induce a facilitatory effect on left IPL through transcallosal disinhibition (50). As such, inhibitory cTBS over the rIPL is a promising method to reduce gesture deficits in schizophrenia. We decided to use the same cTBS protocol for repetitive administration in a larger sample.

#### Social Cognitive Remediation Therapy

Social cognition is a main driver of recovery and functional outcomes (10, 51–53) and mediates the relationship between neurocognition and social functioning in schizophrenia (53). The MATRICS initiative (Measurement and Treatment Research to Improve Cognition in Schizophrenia) of the National Institute of Mental Health (NIMH) identified the most important cognitive domains in schizophrenia (10, 54). These consist of *six neurocognitive domains*: speed of processing (1), attention/vigilance (2), working memory (3), verbal learning (4), visual learning (5) and reasoning/problem solving (6) and *five social-cognitive domains*: emotion processing (1), social perception (2), theory of mind (3), social schemata/knowledge (4) and social attribution styles (5). Therapies focusing on both neurocognition and social cognition are suggested to be a promising approach to help patients with schizophrenia (55–57).

One of the few integrative cognitive remeditation therapies (CRT) covering *all* MATRICS domains is the Integrated Neurocognitive Therapy (INT) (58, 59). This CRT approach takes place in a group setting, including social interactive tasks and computerized neurocognitive exercises (using the Cogpack program). INT showed good feasibility with reduction of positive symptoms, negative symptoms and relapses, as well as, improvements in social cognition, neurocognition, and overall functioning in patients with schizophrenia (56, 60–62). The INT includes four modules, each addressing various cognitive domains: processing speed, attention, and perception of emotions (1); verbal and visual learning, memory, social perception, and theory of mind (2); reasoning, problem solving, and social schema (3); working memory and the ability to attribute appropriate meanings (4). INT focuses on the improvement of both social cognition and neurocognition, with more focus on the latter.

Social cognitive remediation therapy (SCRT) is an approach focusing mainly on social cognition in patients with schizophrenia (58, 63). As of today, two types of SCRTs are being developed: *targeted* and *broad-based* SCRTs. Targeted SCRTs address only the remediation of one specific social cognitive deficit (e.g., facial affect recognition) (64), whereas broad-based SCRTs address multiple social cognitive domains (65). Integrative broad-based SCRTs combine the improvement of social cognitive domains with other therapy techniques, such as Cognitive Behavioral Therapy (CBT) (66), cognitive remediation (67, 68) or social skills training (69–71). It is advisable to use an integrative approach involving both neurocognitive *and* social-cognitive domains, as these lead to greater generalization effects on functional outcome (53, 72, 73). In light of previous studies, we consider integrative broad-based SCRTs a promising treatment to reduce social cognitive impairments and enhance functioning in schizophrenia. Therefore, we decided to use this type of treatment approach for the current study.

## Methods

### Aims and Hypotheses

This study’s main goal is to determine the effects of combined cTBS over rIPL and SCRT on gesture performance and social functioning in patients with schizophrenia. In addition, we aim to investigate neurocognitive, social cognitive, motor, clinical and neural effects of both interventions as secondary outcome measures. As cTBS enhances neuroplasticity and improves use of neural resources (46, 74), we expect administration of cTBS to facilitate SCRT training effects. Additionally, we hypothesize that the expected improvement of gesture performance will have positive effects on functional outcome, as both have been reported to be linked in schizophrenia (20). At the neural level, we hypothesize that the brain activity of patients will converge to the brain activity of healthy controls after both interventions, especially within the praxis network (39, 40).

**FIGURE 1:**
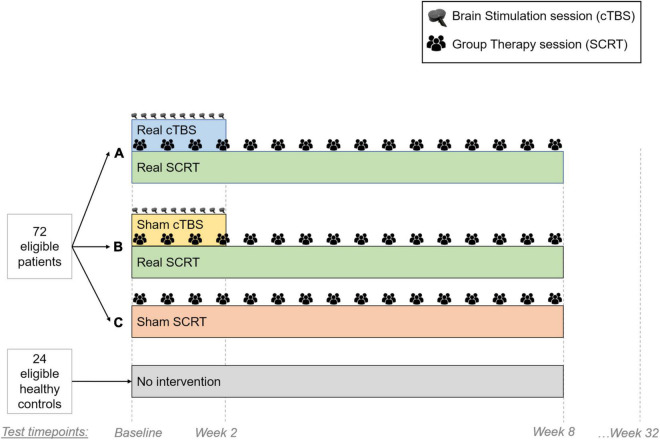
Study Setup. We will randomize 72 schizophrenia patients in three different groups (24 patients per group). Group A will receive real cTBS and real SCRT. Group B will receive sham cTBS and real SCRT. Group C will receive no cTBS and sham SCRT. Both sham treatments are inactive interventions that mimic as closely as possible the real treatments. Group A and B will receive 10 cTBS sessions during the first two weeks of intervention. Additionally, Group A–C will receive 16 SCRT sessions for eight weeks. Outcomes will be measured during four different time points: baseline, after cTBS (week 2), after SCRT (week 8) and follow-up (week 32). We will also include a fourth group consisting of 24 healthy controls, who will be assessed twice (baseline and week 8) without any intervention in between.

This randomized controlled trial (RCT) will include three patient groups (see Figure 1). First, group A will receive real cTBS and real SCRT. Second, group B will receive sham cTBS and real SCRT. Third, group C will receive no cTBS and sham SCRT. In this study, we will also include a fourth group consisting of healthy controls, who will be assessed twice without any intervention in between. We expect the combination of cTBS and SCRT to show positive effects on both primary and secondary outcomes in patients with schizophrenia spectrum disorders. As such, we expect group A to have superior performance over group B and C in gesture performance and functional outcome after intervention (A > B > C).

### Setting and Enrollment

#### Study Design

This will be a three-arm, double-blind, randomized, sham-controlled trial of add-on brain stimulation and SCRT to improve nonverbal communication skills and overall functioning in schizophrenia spectrum disorders. This single-site trial will be conducted at the University Hospital of Psychiatry and Psychotherapy, Bern.

#### Study Population

Patients will be asked for participation at the outpatient departments of the University Hospital of Psychiatry and Psychotherapy, Bern and will be screened with the Structured Clinical Interview for DSM-5 (SCID). Healthy participants will be recruited by word-of-mouth and flyers in public places (e.g., grocery stores, pharmacies, gyms) in Bern. Participants will be eligible for study entry if they meet the criteria listed in Table 1.

**TABLE 1:** Eligibility.

Inclusion criteria
Age between 18 and 65 years
Right-handedness
*Patients only:* diagnosis of schizophrenia spectrum disorders according to DSM-5
**Exclusion criteria**
Substance abuse or dependence other than nicotine
Past or current medical or neurological conditions associated with impaired or aberrant movement, such as brain tumors, stroke, Parkinson’s disease, Huntington disease, dystonia, or severe head trauma with subsequent loss of consciousness.
Epilepsy or other convulsions
History of any hearing problems or ringing in the ears
Standard exclusion criteria for MRI scanning and TMS, e.g., metal implants, claustrophobia
Women who are pregnant or breast feeding or intention to become pregnant during the study
Previous enrollment into the current study
*Patients only:* any TMS treatment in the past 3 months
*Patients only:* any cognitive remediation therapy in the past 2 years
*Controls only:* history of any psychiatric disorder or any first-degree relatives with schizophrenia spectrum disorders.

### Interventions

#### Implementation of Real/Sham Continuous Theta Burst Stimulation

Patients will receive ten cTBS sessions in total during the first 2 weeks of intervention. Each session (sham and real cTBS) will last 17 min consisting of two 44 s stimulations over rIPL separated by a 15-min break. For each stimulation of 44 s, we will apply 801 pulses at 50 Hz over the right IPL at an intensity of 100% of the resting motor threshold. Hence, the entire session will consist of 1,602 pulses. Sham cTBS will be delivered with a placebo-coil without any magnetic emission.

#### Implementation of Real/Sham Social Cognitive Remediation Therapy

Real/sham SCRT will be conducted bi-weekly for 8 weeks, totaling 16 sessions of 90 min. Each group will consist of six to eight patients led by a head-therapist (VC) and a co-therapist (FW). INT-expert (DM) will train both therapists and supervises the course of the sessions.

For this trial, we will tailor an SCRT based on the INT manual (58, 59). This SCRT will include the entire social cognitive part of the original INT (consisting of a minimum of 30 sessions) and two INT neurocognitive modules. As such, we will use psychoeducation and Cognitive Behavioral Therapy for Psychosis (CBTp) techniques. This intervention will have the main goal of improving gesture deficits in schizophrenia and will be separated in five blocks; each including neuro- and social cognitive MATRICS-domains (see Table 2). The first, third and fifth block will focus predominantly on using and interpreting nonverbal cues in social contexts. The second and fourth blocks will address memory and attention, both of which are essential for functioning in social interaction. Three sessions will be dedicated to each block, totaling 15 sessions overall. With each session, the content of the blocks will increase in complexity. A final 16th session will be added, in which the content delivered in all previous five blocks will be summarized.

**TABLE 2:** SCRT therapy contents a trained cognitive processes.

	Introductory session: Psychoeducation topics	Training session: Compensation and restitution sessions with interactive and computerized exercises	Transfer session: Practicing coping strategies in daily life with homework
*Block 1:* *Emotion perception and expression*	Six basic emotions: their function, their expression (e.g., gestures) and their effect on perception/attention	Affect recognition and affect expression	Practicing newly learned affect recognition strategies with their regular environment
*Block 2:* ***Verbal/visual learning and memory***	Short term memory, long term memory and prospective memory	Mnemonic strategies (e.g., chunking, external memory aids such as calendars and post-its)	Practicing familiar and newly learned mnemonic strategies
Block 3: Social perception and theory of mind	Key social stimuli and theory of mind	Observation of social encounters (e.g., with pictures, videos, and role play) and interpretation of social information	Practicing a more fact-oriented social perception (rather than assumptions-based) with their regular environment
Block 4: Working memory	Selective attention, working memory and how to reduce distractibility	Cognitive flexibility, selective attention, ability to inhibit cognitive interference (e.g., with computer tasks)	Practicing newly learned attention-focusing strategies
Block 5: Social schema	Norms and roles on social behavior and use of social knowledge	Social norms and behavioral sequences (e.g., with role play), recognition of own norm-deviating behavior and building strategies to change it if necessary, as well as coping with social stigma	Practicing newly learned social strategies in role play during group therapy and with their regular environment

Each block will start with an introductory session, followed by a training session (i.e., compensation and restitution sessions), and a transfer session (see Table 2). During the introductory sessions, the MATRICS domains will be presented with a great focus on their everyday use and individual self-reference. The introductory sessions include psychoeducation on the respective neurocognitive and social domains. During the training sessions, we will determine patients’ strongest coping strategies, establish solution-oriented awareness, and train cognitive domains with various exercises (e.g., computerized cognitive tasks or role play exercises). The transfer sessions will work on the principle “rehearsal learning” (75, 76), meaning patients repeatedly apply the newly learned strategies in their daily life to restore social cognitive functions. The treatment goal will be to develop more coping strategies and enhance social skills to increase overall functioning. The therapists will follow a script for quality assurance and treatment fidelity.

Sham SCRT will consist of psychoeducation on sleep hygiene, exercise, stress, and music, as well as leisure activities, and mindfulness-based exercises (e.g., raisin meditation or walking meditation in a nearby park in Bern). Patients receiving this group therapy will also benefit from an interactive environment. The main difference with the real SCRT will be that sham SCRT will not include any (social) cognitive training, nor active transfer of skills into daily life.

### Primary and Secondary Outcomes

#### Behavioral and Clinical Assessments

The primary outcome of this study will be hand gesture performance, which will be assessed with the Test of Upper Limb Apraxia (TULIA) (77). TULIA includes 48 items in two domains: Imitation (following demonstration) and pantomime (after verbal command). The test has three semantic categories of gestures: communicative (intransitive), object-related (transitive), or meaningless. Item ratings range from 0 to 5. If the participants make content errors (e.g., perseverations, substitutions), they will get 0–2 points per item. If the participants make spatial or temporal errors, they will get 3–4 points. Perfect performance equals 5 points, which is the maximum score per item. The highest TULIA total score is 240. A cutoff score will be used to measure gesture deficits in schizophrenia with < 210 TULIA total score (17). Gesture performance will be recorded on video and later evaluated by an independent examiner who is blinded to the groups or timing of the assessment. We will assess gesture performance using TULIA at four different time points (see Table 3).

**TABLE 3:** Time-points of assessments.

	Screening	Baseline	Week 2 tests	Week 8 tests	Follow-up
TIME (week)	–4 to 0	0	2	8	32
**Enrollment**					
Eligibility screening	**X**				
Informed consent	**X**				
Allocation	**X**				
**Interventions**					
Real and sham brain stimulation		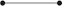			
Real and sham group therapy		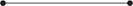			
**Assessments**					
Gesture tests	**X**	**X**	**X**	**X**	**X**
Clinical rating scales	**X**	**X**		**X**	**X**
Community functioning		**X**		**X**	**X**
Neuroimaging		**X**		**X**	

Secondary outcomes will include additional nonverbal communication, cognitive, clinical, and functional assessments, as well as self-reports. Nonverbal communication will be tested with the Profile of Nonverbal Sensitivity (PONS) (78) assessing nonverbal social perception, and the modified postural knowledge task (79, 80) measuring gesture perception. Together, TULIA, PKT, and PONS assess nonverbal communication, as they include the *production*, the *recognition*, and the *interpretation* of gestures. History of symptoms and medication in patients will be assessed with The Comprehensive Assessment of Symptoms and History (CASH) (81). Self-reports, clinical scales, motor scales, cognitive tests and functional assessments are listed in Table 4.

**TABLE 4:** Primary and secondary outcomes.

Assessments		References
Self-reports	Brief Assessment of Gestures (BAG)	(95)
	Self-report of negative symptoms (SNS)	(96)
	The satisfaction with therapy and therapist scale-revised (STTS-R) for group psychotherapy	(97)
Psychopathology	Positive and negative syndrome scale (PANSS)	(98)
	Brief negative symptom scale (BNSS)	(99)
	Thought and language disorder (TALD)	(100)
	Frontal assessment battery (FAB)	(101)
	Bern psychopathology scale (BPS)	(102)
Motor scales	Neurological evaluation scale (NES)	(103)
	Bush Francis catatonia rating scale (BFCRS)	(104)
	Salpêtrière retardation rating scale (SRRS)	(105)
	Unified Parkinson’s disease rating scale (UPDRS)	(106)
	Abnormal involuntary movement scale (AIMS)	(107)
Behavioral tests	Postural knowledge test (PKT)	(79)
	Profile of nonverbal sensitivity (PONS)	(78)
	Coin rotation	(108)
	Test of Upper Limb Apraxia (TULIA)	(77)
Neuro- and social cognition	Mayer-Salovey-Caruso Emotional Intelligence Test (MSCEIT)	(109)
	Digit span backward (DSB)	(110)
	Test of nonverbal Intelligence (TONI)	(111)
	EMOREC-B	(56)
	Hinting task (HT)	(112)
	EmBODY tool	(113)
Functional assessments	Global assessment of functioning (GAF)	(88)
	Social and occupational functioning assessment score (SOFAS)	(114)
	Personal and social performance (PSP)	(114)
	University of California, San Diego performance-based skills assessment brief (UPSA-b)	(115)
	Social level of functioning (SLOF)	(116)

**FIGURE 2:**
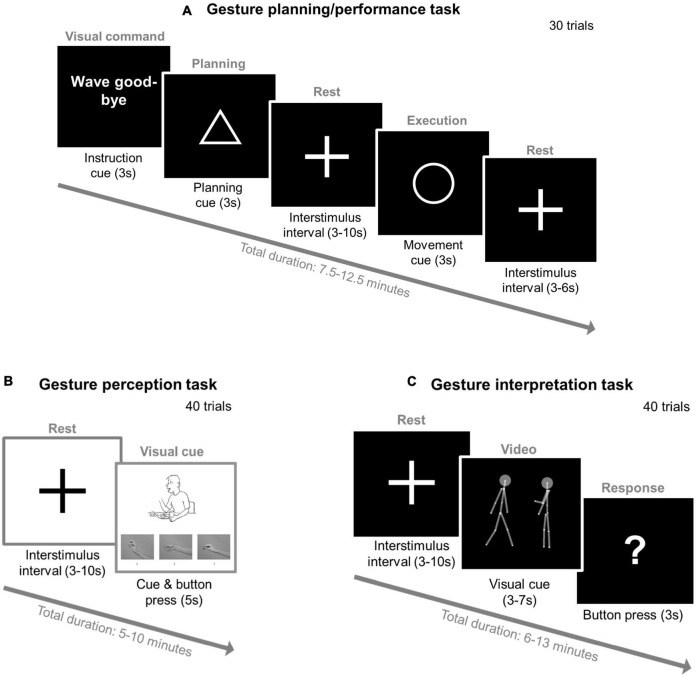
Three fMRI gesture tasks—experimental setup. **(A) Gesture planning/performance task** with the modified Test of Upper Limb Apraxia (TULIA). Participants will be presented with 20 hand gestures in a random order (e.g., wave good-bye) and 10 control sentences with no gesture performance expected (e.g., “the airline flight is delayed”). Each trial will start with an instruction (3 s), followed by a triangle (planning phase: 3 s), then a 3–10 s interstimulus interval with a fixation cross (3–10 s), a circle (execution phase: 3 s), another 3–6 s interstimulus interval, and then a new gesture of the next trial is presented. **(B) Gesture perception task** with the modified Postural knowledge task (PKT). Each trial will start with a fixation cross (3–10 s) followed by a picture (max. 5 s) where the hand of the presented figure will be removed. Participants’ task is to choose the correct hand gesture provided below using button presses of a hand pad inside the scanner (1, 2, and 3) and respond while the picture is still displayed. In the example provided the correct gesture is number two. **(C) Gesture interpretation task** with point-light displays (PLD). Each trial will start with a fixation cross (3–10 s) followed by a PLD video, after which a question mark will be displayed (3 s). The video will include two PLD figures: one on the right side and the other on the left side of the screen. During the video, one PLD will perform a gesture while the other PLD will either follow or imitate these performed gestures. Participants’ task will be to indicate with the button press of a hand pad which of the two PLD figures (left or right) is imitating or following the gestures of the other when the question mark will be displayed. The gray connecting lines and the two gray circles (i.e., PLDs’ heads) are a visual aid and will not be present in the actual experiment.

Outcomes will be measured during four different time points: baseline, after cTBS (week 2), after SCRT (week 8) and follow-up (week 32) (see Table 3). At baseline, week 8 and week 32, the primary and all secondary outcomes will be tested. At week 2, the primary and a few secondary outcomes (i.e., nonverbal communication and clinical assessments) will be measured. Additionally, at baseline and week 8, neuroimaging data will be acquired to test the neural effects of the treatments.

#### Magnetic Resonance Imaging Measurements

Neuroimaging will include structural T1, arterial spin labeling (ASL), diffusion tensor imaging (DTI), blood oxygenation level dependent (BOLD) resting state and fMRI tasks. MRI acquisition will be performed at a 3T SIEMENS MAGNETOM Prisma at the Swiss Institute for Translational and Entrepreneurial Medicine (SITEM), Bern, Switzerland. We will use a 20-channel radio-frequency head coil (Siemens, Germany) for all acquisitions.

##### Functional Magnetic Resonance Imaging and Tasks

We will administer three different fMRI tasks to assess gesture planning/execution, perception, and interpretation (see Figure 2). First, gesture planning and performance will be assessed with an adapted version of the gesture task created by Stegmayer et al. (39) and Kroliczak and Frey (82). In an event related design, participants will be presented with 30 gestures in a random order: 10 meaningless (e.g., “raise your thumb and your index finger”), 10 meaningful gestures (e.g., “wave good-bye”) and 10 control sentences with no gesture performance expected (e.g., “the weather is bad”). Second, we will use the Postural Knowledge Task (PKT) (15, 26, 80) to assess participants’ gesture perception. Participants will be presented with pictures of people performing gestures (20 trials), while the hands executing those gestures are missing. Participants will be asked to choose the correct hand gesture from three choices provided. In the control condition (20 pictures), participants will be asked to indicate if the person performing the gesture is either male or female. Third, we will use the point-light-display (PLD) method to assess dynamic gesture perception. Here, human biological movements will be depicted by 12 point-light dots on the main joints of a human actor in the absence of any other visual characteristics (83–85). For our task, 40 videos of one PLD agent will be shown to perform different communicative gestures while the other PLD agent imitated/followed these performed gestures (26, 86). Participants’ task will be to identify which PLD was imitating/following the other.

### Allocation and Randomization

Interventions will start in blocks as soon as 6–8 participants can be randomized to three groups: Group A, group B or group C (see Figure 1). Patients will not know which treatment they receive; they will be allocated to one of three treatment arms in a blinded manner.

### Statistical Considerations and Plan

Assuming a medium effect size (*f* = 0.15) in a repeated measures ANOVA with moderate-high correlation between time points (0.75), a power of 0.95 and an alpha = 0.05, we will need 72 patients (24 per group). In addition, a control group of 24 healthy subjects will be required. All four groups will be compared pre-to-post with an interval of 8 weeks (see Figure 1). The main effects of the combined interventions on the primary and secondary outcomes will be calculated in a repeated measures design including 3 main time points (Baseline, week 2, and week 8) and three patient groups (A, B, and C). With the latest SPSS and R versions, we also will run additional (partial) correlations, linear regressions, MANCOVAS using covariates (e.g., age, education, and medication).

### Trial Status

Study recruitment started on the 17th December 2019. The first group started with both interventions on the 24th February 2020. The study was impacted by the federal measures against the COVID-19 pandemic. As of June 2022, we recruited 65 patients and 36 healthy controls. We completed baseline assessments with 62 patients and 33 healthy controls. We finished week-8 assessments with 36 patients and 32 controls.

## Discussion

### Relevance of the Current Study

With this study, we will run one of the few prospective, sham-controlled RCTs addressing both gesture deficits and poor functioning in schizophrenia. Ameliorating functional outcome as a means of reintegrating patients into society has become one of the most central goals of psychiatric rehabilitation (87–89). Despite advances in antipsychotic medications and psychotherapies, functional recovery rates of schizophrenia patients have not increased substantially over the last 30 years (90, 91). Schizophrenia is not only a major burden for patients, but also a great economic strain for society, as patients with schizophrenia make heavy use of inpatient services and require financial support. It is urgent to develop psychiatric treatments that help patients to maintain interpersonal relationships and facilitate independent living. As gesture deficits in schizophrenia are highly correlated to overall functioning in daily life (20) and effective therapies reducing nonverbal communication deficits are lacking, it is clear that we need to establish interventions that tackle both (46, 92).

### Advantages

The design of this RCT encompasses numerous advantages. The main strength of our study is the *combination* of two interventions; coupling brain stimulation with group therapy may exert cumulative therapeutic effects in gesturing and functioning in schizophrenia (20). Additionally, cTBS enhances neuroplasticity in schizophrenia patients (46, 74) and thus, may facilitate SCRT training effects. Thanks to the two comparators (sham cTBS and sham SCRT) we can evaluate the therapeutic effects of both interventions on behavioral, clinical, and neural levels. Also, we may find insights to predictors of treatment outcome. Further, the RCT design allows elimination of selection bias in treatment assignment and enables blinding from assessors and participants. Moreover, by collecting detailed demographic information over time, we will be able to correct for age, gender, education and antipsychotic medication dosage. Longitudinal data allows exploration of change in our patients, and thus, gives insight on the longevity of treatment effects. For example, our design may shed light on factors that are essential for the transfer of coping strategies. Most importantly, we are running one of the very few RCT on SCRT to involve acquisition of neuroimaging data before and after intervention. We can directly compare neural alterations to potential behavioral changes after the interventions. If this study proves to be successful it has the potential to be beneficial for other psychiatric disorders, which exhibit similar socio-cognitive dysfunctions such as depression (93, 94).

### Limitations

This RCT entails a few limitations. First, we cannot eradicate the influence of medication; most patients suffering from schizophrenia are on antipsychotic treatment and other medication (e.g., blood pressure suppressants, antidepressants). However, for antipsychotic medication, we will control for Olanzapine equivalents in our statistical analyses. Second, we cannot eliminate gains linked to repeated test exposure as a result of retaking the same test under comparable conditions (i.e., retest effects). We counter these effects by randomizing the order of our test items for most of our assessments. Additionally, the time between most of our assessments makes training effects quite unlikely (i.e., 8 weeks, 32 weeks). Third, some of the data we collect are self-reports. Due to the subjective nature of self-reports, their psychometric aspects (e.g., reliability and validity) can be questionable or subject to biases (e.g., response bias). However, self-reports are necessary. For example, it is essential to compare patients’ subjective symptoms before and after intervention to find out if we are helping them. Fourth, another concern is the generalizability of the trial results. Participation in our study is only appropriate for a subgroup of schizophrenia patients, as it consists of 30 meetings in 3 months. As such, we can only include stable outpatients with good time management, commitment and tolerance to additional stress. This might cause the data collection to become arduous and dropout rates to be high.

## Conclusion

In summary, this study explores the combination of cTBS and SCRT to improve gesture skills and social functioning in schizophrenia. This research project is of great importance as treatment options alleviating nonverbal communication deficits are clearly lacking. If this study proves to be successful, it has the potential to change the course of current treatment methods and greatly improve social functioning, as well as the quality of life of patients with schizophrenia.

## Ethics Statement

The studies involving human participants were reviewed and approved by the Kantonale Ethikkommission Bern (KEK). The patients/participants provided their written informed consent to participate in this study.

## Author Contributions

VC contributed to recruits participants, organizes the study procedures, conducts assessments, carries out both interventions, and drafted the manuscript. AP supervised the study procedures. DM trained the head therapist and co-therapists in SCRT and supervised the SCRT sessions. SW designed the study, obtained funding and ethics approval, wrote the study protocol, and registered the study at the clinical trials. All authors discussed and critically revised the manuscript.

## Conflict of Interest

SW was received honoraria from Mepha, Neurolite, Janssen, Lundbeck, Otsuka, and Sunovion. The remaining authors declare that the research was conducted in the absence of any commercial or financial relationships that could be construed as a potential conflict of interest.

## Publisher’s Note

All claims expressed in this article are solely those of the authors and do not necessarily represent those of their affiliated organizations, or those of the publisher, the editors and the reviewers. Any product that may be evaluated in this article, or claim that may be made by its manufacturer, is not guaranteed or endorsed by the publisher.

## Abbreviations

ASL: arterial spin labeling
BOLD: blood-oxygen-level-dependent imaging
CBTp: Cognitive Behavioral Therapy for Psychosis
cTBS: continuous theta burst stimulation
DTI: diffusion tensor imaging
fMRI: functional magnetic resonance imaging
IFG: inferior frontal gyrus
iTBS: intermittent theta burst stimulation
IPL: inferior parietal lobule
MATRICS: Measurement and Treatment Research to Improve Cognition in Schizophrenia
MRI: magnetic resonance imaging
PKT: postural knowledge test
PLD: point-light-display
PONS: profile of nonverbal sensitivity
RCT: randomized controlled trial
rIPL: right inferior parietal lobule
rTMS: repetitive transcranial magnetic stimulation
SCRT: social cognitive remediation therapy
SMA: supplementary motor area
TULIA: Test of upper limb apraxia.
